# Simultaneous Analysis of All SNPs in Genome-Wide and Re-Sequencing Association Studies

**DOI:** 10.1371/journal.pgen.1000130

**Published:** 2008-07-25

**Authors:** Clive J. Hoggart, John C. Whittaker, Maria De Iorio, David J. Balding

**Affiliations:** 1 Department of Epidemiology and Public Health, Imperial College, London, United Kingdom; 2 Non-Communicable Disease Epidemiology Unit, London School of Hygiene and Tropical Medicine, London, United Kingdom; Queensland Institute of Medical Research, Australia

## Abstract

Testing one SNP at a time does not fully realise the potential of genome-wide association studies to identify multiple causal variants, which is a plausible scenario for many complex diseases. We show that simultaneous analysis of the entire set of SNPs from a genome-wide study to identify the subset that best predicts disease outcome is now feasible, thanks to developments in stochastic search methods. We used a Bayesian-inspired penalised maximum likelihood approach in which every SNP can be considered for additive, dominant, and recessive contributions to disease risk. Posterior mode estimates were obtained for regression coefficients that were each assigned a prior with a sharp mode at zero. A non-zero coefficient estimate was interpreted as corresponding to a significant SNP. We investigated two prior distributions and show that the normal-exponential-gamma prior leads to improved SNP selection in comparison with single-SNP tests. We also derived an explicit approximation for type-I error that avoids the need to use permutation procedures. As well as genome-wide analyses, our method is well-suited to fine mapping with very dense SNP sets obtained from re-sequencing and/or imputation. It can accommodate quantitative as well as case-control phenotypes, covariate adjustment, and can be extended to search for interactions. Here, we demonstrate the power and empirical type-I error of our approach using simulated case-control data sets of up to 500 K SNPs, a real genome-wide data set of 300 K SNPs, and a sequence-based dataset, each of which can be analysed in a few hours on a desktop workstation.

## Author Summary

Tests of association with disease status are normally conducted one SNP at a time, ignoring the effects of all other genotyped SNPs. We developed a computationally efficient method to simultaneously analyse all SNPs, either in a genome-wide association (GWA) study, or a fine-mapping study based on re-sequencing and/or imputation. The method selects a subset of SNPs that best predicts disease status, while controlling the type-I error of the selected SNPs. This brings many advantages over standard single-SNP approaches, because the signal from a particular SNP can be more clearly assessed when other SNPs associated with disease status are already included in the model. Thus, in comparison with single-SNP analyses, power is increased and the false positive rate is reduced because of reduced residual variation. Localisation is also greatly improved. We demonstrate these advantages over the widely used single-SNP Armitage Trend Test using GWA simulation studies, a real GWA dataset, and a sequence-based fine-mapping simulation study.

## Introduction

The ideal analysis of a genome-wide association (GWA) study for a complex disease would involve analysing all the SNP genotypes simultaneously to find a set of SNPs most associated with disease risk. Such an analysis can improve performance over single-SNP tests, since a weak effect may be more apparent when other causal effects are already accounted for, but also because a false signal may be weakened by inclusion in the model of a stronger signal from a true causal association. To date, analysing all SNPs simultaneously has seemed infeasible, since current GWA platforms can type over one million SNPs, and even larger variable sets may not be far away as genome-wide re-sequencing advances.

We exploit recent advances in stochastic search algorithms [1],[2] to develop a computationally efficient tool to simultaneously analyse *k* SNPs typed in *n* individuals for association with case-control status, where *k* » *n*. We formulate the problem as variable selection in a logistic regression analysis that includes a covariate for each SNP. Our aim is to find a subset of SNPs (a “model”) that best explains the case-control status subject to a specified error rate. The number of possible models is 2*^k^* and since *k* is typically of the order of 10^6^, classical methods such as forward-backward variable selection are computationally expensive and are liable to find sub-optimal modes [3]. Bayesian stochastic search methods have been used to tackle variable selection problems, typically using the “slab and spike” prior formulation [4]. Inference can be made from these models using Markov chain Monte Carlo (MCMC) [5],[6] and this methodology has been extended to the case of more variables than observations [7],[8]. Similar methods have been proposed for the analysis of SNP data [9],[10], which again utilised MCMC. However, despite design of the MCMC algorithms to minimise computational time, these methods are too slow to deal with the size of problem presented by modern SNP chips. Furthermore, these methods have dealt with the computationally easier problem of a continuous outcome.

We assign continuous prior distributions with a sharp mode at zero, often referred to as “shrinkag” priors, to the regression coefficients. Our approach is Bayesian-inspired rather than fully Bayesian, since we seek only the posterior mode(s) rather than the full posterior distribution of the regression coefficients. If the signal of association at a SNP is weak or non-existent, the posterior mode for the corresponding covariate will remain at zero. By using continuous shrinkage priors the resulting posterior density is continuous and can thus be maximised using standard algorithms. Our stochastic search maximisation algorithm seeks the (small) subset of SNPs for which the posterior mode is non-zero, corresponding to a signal of association that is strong enough to overcome the prior preference for zero effect. The algorithm can be set to include only additive effects, or it can also consider dominant and recessive terms: only one of these terms is permitted to be non-zero.

**Figure 1:**
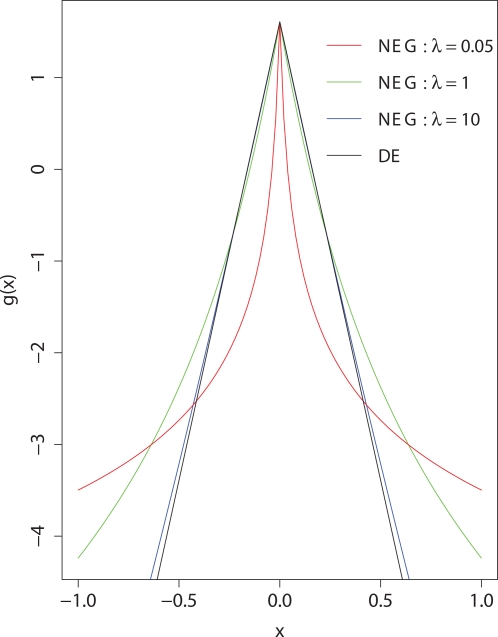
Logarithms of NEG and DE densities. Fixed to have the same density at the origin.

We consider two shrinkage prior distributions, the Laplace, or double exponential distribution (DE) and a generalisation of it, the normal exponential gamma distribution (NEG), which has a sharper peak at zero and heavier tails, Figure 1. The sharp peak of the NEG at zero favours sparse solutions which is preferable for variable selection when we believe that there are few true causal variables. Further, the heavy tails result in variables being minimally shrunk once included in the model. The NEG is characterised by a shape and a scale parameter. The smaller the shape parameter the heavier the tails of the distribution and the more peaked at zero. Conversely as the shape parameter increases the NEG approaches the DE. For both prior distributions we obtain an explicit expression for the approximate type-I error of our method, so that it can be calibrated without recourse to permutation techniques.

With both the NEG and DE prior for *n*<*k*, the posterior density can be multi-modal [11] and the mode identified by each run of our algorithm depends on the initial values of the parameters and the order in which they are updated. We implement multiple runs of the algorithm, always starting with all regression coefficients equal to zero but permuting the order in which they are updated, and report the highest of the modes identified. Our checks using more extensive searches in test datasets indicate that typically the largest mode identified in our search corresponds to a model that is very similar to the global optimum model, differing for example in which of two highly-correlated SNPs is included. We demonstrate this in our analysis of a real GWA study and show how the multiple modes found can be utilised to infer a group of SNPs that identify the same signal.

As a consequence of modelling all SNPs simultaneously, a SNP will only be included in the model if it significantly improves prediction of case-control status beyond that obtained from the SNPs already included. Thus a SNP with strong marginal effect can be overlooked by our analysis if other SNPs better explain most of its effect. We typically find that our analysis returns only the best SNP characterising the effect of a single detectable causal variant, and when multiple SNPs in close proximity are selected this is an indication of multiple distinct causal variants. Thus, the number of SNPs in the best fitting model gives an estimate of the number of causal variants. This feature of the method also makes it suitable for fine mapping using dense SNP sets, such as those that can arise from imputation methods or re-sequencing, in contrast with single-SNP analyses in which many tightly-linked SNPs may show signs of association, leaving open the problem of locus refinement.

Haplotype and interaction effects could be readily implemented using our approach, but these would substantially increase the size of the model space to be explored for genome-wide datasets and we have not pursued these possibilities here. Our software deals with quantitative phenotypes, but here we focus on main effect terms for case-control phenotypes with up to half a million SNPs, and demonstrate that our method improves on single-SNP analyses in terms of false-positive rate, power and interpretability.

## Results

### Main Simulation Study

Our main simulation study used the FREGENE software [12] to simulate 20 Mb of sequence data in a population of 10 K individuals with mutation, cross-over and gene-conversion rates similar to those in humans [13]. From this population we sampled 500 case-control data sets each with six causal variants and 1,000 cases and 1,000 controls. For each simulation we added a further nineteen 20 Mb chromosomes devoid of causal variants. Thus, in effect we analysed 400 Mb genomes consisting of twenty equal-length chromosomes, with all the causal variants concentrated on one chromosome. Marker SNPs were sampled to give an approximately uniform minor allele frequency (MAF) distribution with SNPs spaced on average every 5 Kb, giving 80 K SNPs per data set. The selection ignored causal status, so that the marker SNPs usually included few if any of the causal SNPs.

The above data sets were analysed using (i) our algorithm with an NEG shrinkage prior, (ii) our algorithm with a DE shrinkage prior, and (iii) the Armitage trend test (ATT). When using the NEG and DE we standardised the genotype data to have mean zero and variance one. The ATT is the natural univariate comparison for our multivariate method being a score test for a regression coefficient in a logistic regression model [14] and we show in Text S1 that our search procedure, when applied univariately to standardised data, is asymptotically equivalent to the ATT. Detailed analyses on a subset of the data identified 0.05 as the most suitable value of the NEG shape parameter *λ* for the selection of truly causal variants; smaller values gave rise to computational problems. With *λ* = 0.05 the heavy tails of the prior density (Figure 1), reflect little prior knowledge of effect sizes. However, standardising the genotypes has the effect of incorporating a prior belief that effect sizes may be larger at alleles with smaller MAF [15]. The per-SNP type-I error rate was set at *α* = 10^−5^ for all three analyses; see Methods for setting the DE and NEG parameters to achieve this. The results for the NEG and DE were based on the highest posterior mode found from 100 permutations of the search order using the optimisation algorithm described in the Methods.

The definition of true and false positives can be problematic when, as here, the causal variant is typically not included among the SNPs analysed. In practice, when a significant result is obtained all SNPs in strong linkage disequilibrium (LD) with it are considered as potential sources of the positive signal. For unlinked SNPs with uniform MAF distribution and typed in 2 K individuals, the upper 99.9% percentile of the null distribution of *r* ^2^ is about 0.005, and so most SNPs having *r* ^2^>0.005 with a causal variant are in some sense true associations. However, due to the variable pattern of LD in the human genome, SNPs showing 0.005<*r* ^2^<0.05 with a causal variant can sometimes be hundreds of Kb distant from it, and are likely to be difficult to replicate. Therefore we chose to classify a positive signal as “true” if it has *r* ^2^>0.05 with any causal SNP. Furthermore, a cluster of tightly linked false positives might be considered as essentially just one false positive, and in Table 1 false positives were only counted if they were further than a specified distance (between 0 Kb and 100 K) away from any other false positive that had already been recorded.

**Table 1:** Main simulation study: the results shown are summed over the 500 datasets each with 6 causal variants; a causal variant is “tagged” if ≥1 selected SNP has *r* ^2^>0.05 with it.

Method	SNPs selected	Causal SNPs tagged	False positives minimum separation (Kb)			
			0	20	40	100
NEG	2097	1576	368	368	368	366
DE	2622	1501	297	277	276	271
ATT	6810	1554	696	536	486	441

A feature of both the NEG and DE results is the reduction in the number of false positives relative to the ATT, despite the fact that the type-I error rate was set to be the same for all analyses (Table 1). This reflects one of the principal effects of analysing SNPs simultaneously: the signal at a SNP that shows spurious association when analysed singly is often weakened by inclusion in the model of true positives, which may or may not be tightly linked with it.

**Figure 2:**
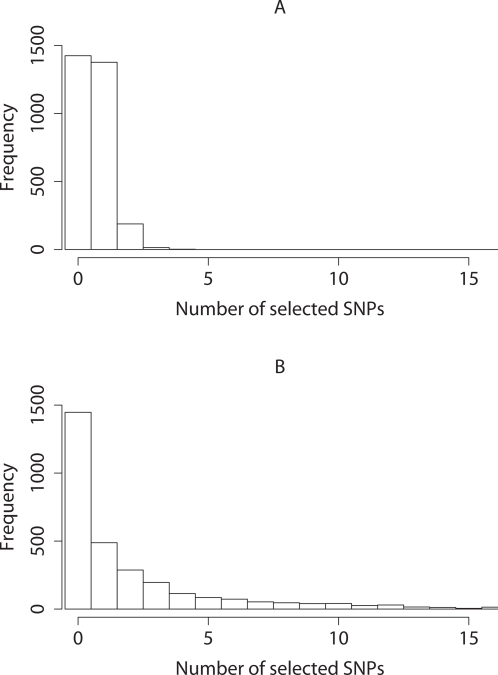
Main simulation study. Histograms of the number of selected SNPs tagging (at *r* ^2^>0.05) each causal SNP for (A) NEG and (B) ATT analyses.

If several SNPs are mutually in high LD, typically at most one of them will be included in the model. Thus the NEG analysis picked 2,097 SNPs over the 500 data sets, many fewer than the 6,810 SNPs selected using the ATT (Table 1). If a causal SNP is detected by the NEG method then typically (87% of the time) it will be tagged by just one selected SNP (Figure 2). In contrast ATT often picks multiple SNPs for each causal variant and picks one SNP just 31% of the time. The higher number of false positives for ATT can be attributed in part to the way the ATT picks many more SNPs per causal variant than the NEG or DE; some of these are remote from, and in low LD with, the nearest causal variant, and fail to reach our threshold of *r* ^2^>0.05 for useful tagging. However, the fact that ATT selects many more SNPs than DE or NEG can spuriously inflate its true power, because some of these additional significant SNPs will by chance be in LD with one or more causal variants. In the case of several causal variants, the ATT may produce what appears to be a single signal.

Of the 3,000 causal SNPs in the simulations, 1,402 are detected by both the NEG and ATT analyses, 54 are only detected by NEG and 32 are only detected by ATT. Although this difference in empirical power is small (0.7%), a *p*-value for the null hypothesis that the NEG and ATT are equally powerful is 0.011 (binomial probability of ≤32 successes, given 86 trials and success probability 0.5). Moreover, the NEG empirical power equals or exceeds that of ATT for all six combinations of MAF and allelic risk ratio (Table 2). Thus we have evidence of improved power of NEG over ATT, in addition to its lower false positive rate.

**Table 2:** Main simulation study: numbers of causal SNPs tagged, out of the 500 for each MAF and risk ratio.

Method	MAF and allelic risk ratio					
	15%		5%		2%	
	1.4	1.5	1.8	2.2	2.5	3.0
NEG	252	360	209	370	146	239
DE	233	347	194	366	135	227
ATT	244	353	209	370	143	235

DE shows fewer false positives than NEG, but it tags fewer causal SNPs even though it selects 2,622 SNPs, many more than NEG (Table 1). The lighter tails of the DE distribution in comparison with the NEG result in informative variables being shrunk closer to zero. This can result in other correlated SNPs being brought into the model to explain the full effect of the over-shrunk SNP coefficients.

Our preliminary analyses varying *λ* showed that as *λ* increased the false positive rate decreased but the power also decreased and in doing so approached the results obtained with the DE prior. With *λ* = 10 the results were very similar to the DE results.

### Null Simulation

To validate our type-I error rate approximation, and to assess the effect on type-I error of allowing for dominant and recessive effects, we permuted the case-control status in one of the 80 K data sets to generate 1,000 data sets representing samples from the null of no genetic effects. The resulting per-SNP type-I error rates of the NEG, DE and ATT methods are shown in Table 3. False positives were only counted if they were further than 20 Kb away from any other false positive that had already been recorded, however the results were the same when the minimum separation was 100 Kb. The type-I error rate was highest for ATT although the differences are not significant. All three analyses result in noticeably fewer false positives than the nominal rate of *α* = 10^−5^. Because of LD between SNPs it is not easy to decide if this difference is significant, but it suggests that our type-I error approximation is as conservative as that of the ATT, which is based on the 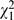 approximation.

**Table 3:** Null simulation: empirical per-SNP type-I error rates from 1,000 permutations of case-control labels of 2 K individuals genotyped at 80 K SNPs.

Method	Error rate (per million SNPs)	
	Additive only	Additive, dominant and recessive terms
NEG	6.44	12.8
DE	6.39	12.7
ATT	6.48	-

In each case the nominal per-SNP type-I error rate for the additive-only model was 10^−5^ ( = 10 per million SNPs).

When dominant and recessive effects are considered in addition to additive terms, the false positive rate approximately doubles. Thus parameter settings that control the type-I error at 2.5% for additive effects will approximately control the type-I error at 5% when dominant and recessive effects are also included. Recall that our simulations generated an approximately uniform MAF distribution of the marker SNPs, and this result may vary with different MAF distributions.

### Whole Genome Simulation Study

We also generated a data set corresponding to a genome-wide association study consisting of 480 K SNPs, derived from 120 independent 20 Mb chromosomes using the same SNP ascertainment strategy as used in the previous simulation. We chose one 20 Mb chromosome to have ten causal variants each with MAF of 15% and allelic risk ratio of 2. This disease model is unrealistic but was chosen to permit detection of the majority of the causal variants and thus make general comments on the relative merits of the NEG and ATT in one simulation.

We analysed this data set as before using the NEG and ATT but with a significance threshold of *α* = 5×10^−7^ [16]. As before, we chose the highest posterior mode from 100 permutations of the search order.

**Figure 3:**
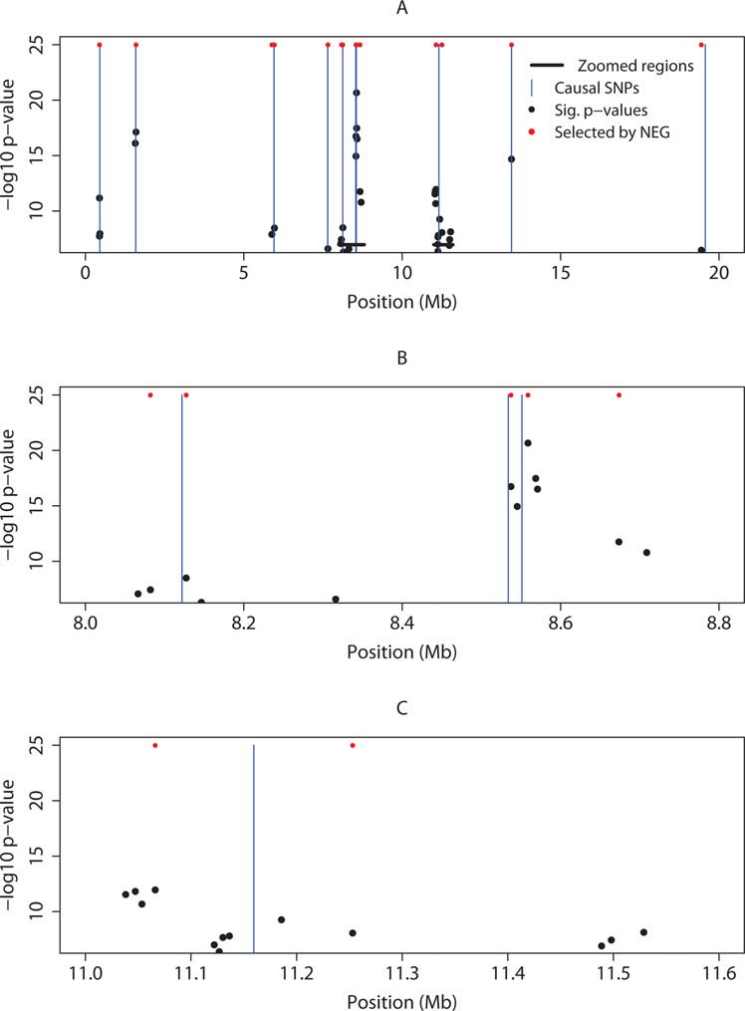
GWA simulation. (A) locations of the ten causal variants (vertical blue line) on the 20 Mb chromosome; also shown are the SNPs selected by NEG (red dots), and the SNPs with ATT *p*-value 5×10^−7^ (black dots) plotted against −log_10_ (*p*-value). (B) and (C) show zooms of two sub-intervals of (A).

Figure 3(A) shows the locations along the 20 Mb chromosome of the ten causal variants, as well as all the SNPs selected by the NEG and ATT analyses. Both methods have detected all ten causal variants, however the NEG analysis selected just 14 SNPs, whereas the ATT identified 35 significant SNPs. We see in Figure 3B that the NEG analysis has improved localisation, it ignores SNPs remote from causal variants that were significant under the ATT, in particular at 8.3 Mb, and has selected SNPs as close to the causal variant as the ATT has. In Figure 3C the improvement in localisation is less clear, the ATT has selected SNPs closer to causal variant, but at the expense of selecting many more SNPs. In particular the ATT has selected SNPs at 11.5 Mb, about 400 Kb from the causal variant.

### Re-Sequencing Simulation Study

Fine mapping of causal variants currently uses very high density markers, obtained either directly from resequencing or from imputation following limited resequencing or from high-density SNPs in public databases [17],[16],[18]. To illustrate the utility of our method for the analysis of imputed and/or sequence data we took the simulated 20 Mb sequences of 10 K individuals used in the previous analyses, which had 192 K polymorphic sites, and sampled 10 case-control datasets using the same sample sizes and disease model as used in the main simulation. All polymorphic sites were included in our analyses. The data sets were analysed with the NEG and ATT with a per-SNP false positive rate of *α* = 10^−5^.

**Figure 4:**
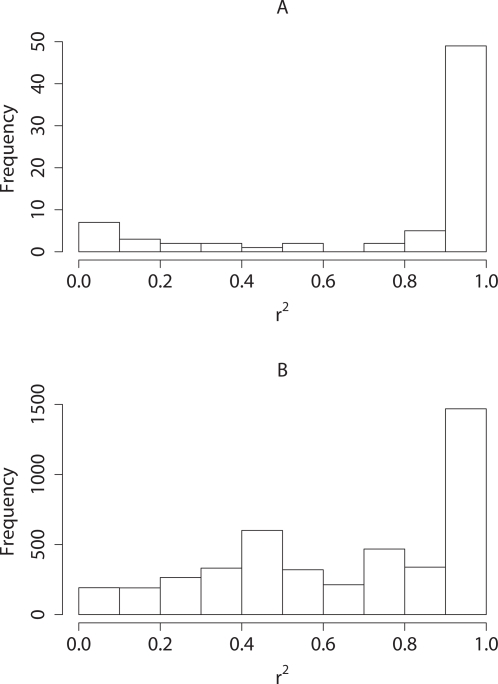
Re-sequencing simulation. Histograms of the maximum *r* ^2^ for each selected SNP with a causal variant for (A) NEG and (B) ATT analyses.

The ATT and NEG analyses showed similar power over the ten sequence-level datasets. Both methods detected 54 of the 60 causal variants with *r* ^2^>0.3, five causal SNPs were missed entirely by both methods and one causal SNP was tagged by both methods at *r* ^2^≈0.01. However, NEG showed markedly better localisation than ATT. Figure 4 shows the distribution of the highest *r* ^2^ value for each selected SNP with a causal variant using the two methods. The NEG selected just 64 SNPs in comparison with 599 selected by the ATT, and a greater proportion of the selected SNPs were in high LD with a causal variant. Of the 60 causal variants, only nine were tagged twice by NEG, in contrast, it is evident from Figure 4 that the ATT often multiply tags causal SNPs. In no simulation did a SNP selected by NEG tag two causal SNPs.

The NEG analysis identified two false positive SNPs at the less stringent *r* ^2^ = 0.01 threshold for tagging a causal SNP. The ATT analysis generated 14 false positives at this threshold; 11 of these SNPs spanned a 230 Kb region including one of the NEG false positives, while two spanned a 103 Kb region including the other NEG false positive. The 14th ATT false positive had *r* ^2^ = 0.009 with a causal variant.

### Analysis of Type 2 Diabetes GWA Data Set

From a genome-wide scan on 694 type 2 diabetes cases and 654 controls [19], we reanalyse here genotype data from the Human Hap300 BeadArray, but not the Human Hap100 BeadArray. After removing SNPs with Hardy-Weinberg equilibrium *p*-value <10^−3^ or with call rate <0.95, there were 300,535 SNPs analysed.

In the original analysis [19], SNPs were tested for additive, dominant and recessive effects and 42 were significant (permutation *p*-value <5×10^−5^), tagging 32 distinct loci (defined here to denote a 1 Mb flanking region). These SNPs, together with 15 SNPs identified using the Human Hap100 BeadArray, were carried forward to a replication analysis using 2,617 cases and 2,894 controls [19] that confirmed eight SNPs tagging five loci.

Our NEG reanalysis used *λ* = 0.05, while *γ* was set such that *α* = 2.5×10^−5^ if additive effects only were considered, thus approximately controlling the type-I error rate at 5×10^−5^ for our actual analysis which also considered dominant and recessive terms. The resulting best-fitting model included 26 SNPs, tagging 25 distinct loci including the five previously-replicated loci (Table 4). Four of our SNPs matched those previously reported while the fifth locus had been tagged by Human Hap100 BeadArray SNPs not included in our reanalysis, but instead we identified rs729287 only 20 Kb distant.

**Table 4:** SNPs included in the best-fitting model for association with type 2 diabetes from the NEG analysis of Human Hap300 BeadArray genotype data that were validated in a second stage analysis [19].

SNP	Chromosome	Position	Closest gene	Model
rs13266634	8	118,253,964	SLC30A8	Dominant
rs7923837	10	94,471,897	HHEX	Additive
rs7903146	10	114,748,339	TCFL2	Additive
rs7480010	11	42,203,294	LOC387761	Dominant
rs729287	11	44,236,666	EXT2	Dominant

We also looked at the seven other best-fitting modes with posterior density within a factor of 10 of the maximum. These modes included 29 unique SNPs. The three extra SNPs in this combined list not included in the best fitting mode were all within 50 Kb of SNPs included in the best fitting mode. In modes which included one of these extra SNPs, the SNP close by was not included. Thus, examining sup-optimal modes can identify alternate SNPs tagging the same causal variants, which can be useful to include in follow-up genotyping when some redundancy is beneficial, or to consider alternative possibilities for the SNP in strongest LD with the causal variant. The SNPs tagging the five previously replicated loci were included in all seven modes suggesting that these are the best tagging SNPs for the causal loci.

These results are consistent with the conclusions from our simulation study: we captured the same significant loci as the single-SNP analysis but at the cost of many fewer false positives. In addition, our NEG analysis has picked one SNP from each of the replicated loci, suggesting that there is just one distinct causal variant in each locus tagged by the genotyped SNPs.

## Discussion

Our NEG shrinkage-based algorithm provides a computationally-efficient tool for the simultaneous analysis of either genome-wide SNPs or resequencing or hyper-dense SNPs from large regions. The NEG analysis improves on the single-SNP ATT analysis, most notably in terms of false positives, and also in terms of power. It is also superior to the DE analysis in terms of of power, at the expense of a higher false positive rate. The NEG method typically selects one SNP for each causal variant and thus gives a measure of the number of underlying causal SNPs genotyped in the data set under study, as well as improving localisation in comparison with the ATT.

The advantages of the NEG analysis are even greater for sequence or very-high density genotype data, such as can be obtained via imputation: it identifies a much smaller subset of SNPs without a reduction of power, and tags the causal variants with higher *r* ^2^ on average. This reflects the natural advantage of a regression-based approach when causal variants are included in the analysis rather than merely tagged by markers.

Significant SNPs from a GWA are usually genotyped in another sample. With cheaper genotyping experimenters may be able to afford to replicate more SNPs than the minimal set suggested by the NEG or DE methods. Candidates for redundant/alternative SNPs can be obtained by considering local modes found by our algorithm. A full Bayesian analysis such as that suggested in [20] would also be possible if limited to a subset of SNPs, and would explore the posterior distribution more completely for that SNP-set.

Since we take a regression approach it would be straightforward to include other individual level covariates such as age and sex, as well as covariates to control for population stratification such as eigenvectors from a principal component analysis [21]. Our software can analyse quantitative traits and could be extended to search for haplotype or interaction effects. In the latter case the size of the model space would need to be reduced, perhaps by a strategy of seeking interactions of all SNPs with those SNPs showing marginally significant association. There is growing interest in predicting phenotypes from genotypes in both human genetics [22] and livestock genetics, in which there is interest in predicting breeding values [9]. Since our method is regression based and considers all SNPs simultaneously and will thus account for the LD between SNPs [23], it can also address this application; a weaker significance threshold is often considered appropriate for prediction rather than SNP selection.

Software can be downloaded from http://www.ebi.ac.uk/projects/BARGEN/.

## Methods

### Shrinkage Priors

For each regression coefficient we assign independent shrinkage priors with a density that is sharply peaked at zero. The DE is a one-parameter distribution that is widely used as a shrinkage prior [1]. It can be represented as a scale mixture of a normal distribution:
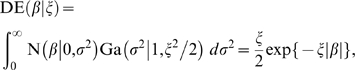
(1)where N(*a*,*b*) is the normal density with mean *a* and variance *b* and Ga (*c*,*d*) is the gamma density with shape parameter *a* and scale parameter *b*.

The normal exponential gamma distribution [2] is a generalisation of the DE that has the following scale mixture representation:
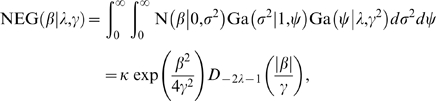
(2)where *λ* and *γ* can be interpreted as shape and scale parameters respectively, *κ* is the integrating constant and *D* is the parabolic cylinder function [24]. We can see from (1) and (2) that the NEG can be generated by sampling from a DE distribution with parameter drawn from a gamma distribution. There is a fast algorithm for computing *D* and its derivatives [25], and Fortran code is available from http://jin.ece.uiuc.edu/routines/routines.html.

As *λ* and *γ* both increase such that 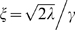 remains constant, the NEG converges to the DE distribution with parameter *ξ*. Figure 1 shows the log densities of the DE and three NEG distributions, all with the same density at zero. From the plot we see that as *λ* decreases the NEG density is steeper near zero and flatter elsewhere, thus shrinking non-zero coefficients less than the DE. For further details of the NEG see Text S1.

### The Optimisation Algorithm

We seek to maximise the posterior density *p* (*β* | ***x***,***y***) over *β* = (*β*_1_,…, *β_k_*), where *x* = (*x*_11_,…,*x_nk_*) is the normalised genotype data and y = (*y*_1_,…,*y_n_*) denotes the case-control status coded as 1 for cases and −1 for controls. Taking logarithms in Bayes Theorem we can write
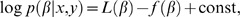
(3)where *L* denotes the log-likelihood for the logistic regression model and *f* is minus the log-prior density. The negative sign is introduced to allow *f* to be interpreted as a penalty function, and so our estimation procedure can be thought of as maximising a penalised log-likelihood. With the DE prior, the maximisation of (3) is equivalent to the Lasso procedure [26]. The EM algorithm has been used for the analogous optimisation problem for linear regression [2] but we found it to converge slowly for binary regression. Instead we use the CLG algorithm [27] which optimises each variable in turn, making multiple passes over the variables until a convergence criterion is met. This algorithm has been implemented for the logistic regression model [1], but not previously with the NEG prior.

There is no closed form solution for the univariate optimisation problem in logistic regression, but Newton's method can be applied using the formula
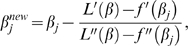
(4)where each ′ denotes a derivative with respect to *β_j_*, and
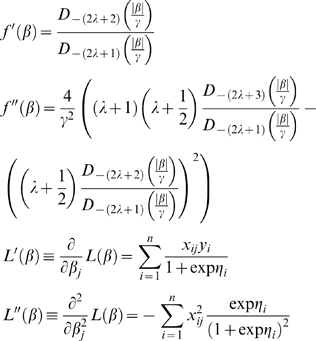
(5)where 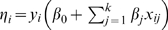. See Text S1 for justifications.

We avoid taking large steps by replacing the *L*″ with an upper bound [1]. We also disallow any update proposed according to (4) that would change the sign of *β*: instead, 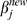 is set to zero. When *β_j_* = 0 the algorithm attempts an update in both directions, taking limits as *β_j_* approaches zero from above and below, and accepting the move if 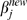 is, respectively, positive or negative. Since the denominator in (4) is always negative at *β_j_* = 0, and *f* is symmetric about zero so that *f*′(0^+^) = −*f*′(0^−^), where 0^+^ and 0^−^ denote the limits from above and below, it follows that a move of *β_j_* away from the origin occurs whenever
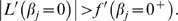
(6)


The calculation of *L*′ involves a sum over all individuals and is computationally expensive. Moreover, recall that the ′ denotes derivative with respect to *β_j_* and so *L*′ is required for each *j*. However, computationally-fast upper and lower bounds for *L*′ can be derived (see Text S1), which in conjunction with (6), determine whether a move from *β_j_* = 0 is possible. Checking this bound avoids the necessity to compute *L*′ for all but a small proportion of values of *j*.

### Assigning Prior Parameters to Control Type-I Error

From (6) we can derive an explicit approximation for the type-I error rate of our procedure. We reject the null if the posterior mode is not at *β* = 0. By standardising the genotype data to have mean zero and variance one, the type-I error probability is the same for each SNP, regardless of MAF. By writing |*L*′(*β*)| in terms of 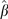, the maximum likelihood estimate of *β*, and assuming asymptotic normality of 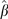, the per-SNP type-I error rate will be α if
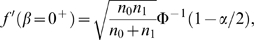
(7)where *n*_0_ and *n*_1_ are the numbers of cases and controls and Φ^−1^ is the inverse normal distribution function; see Text S1 for the derivation. To maintain the same type-I error the prior must be chosen such that the penalty *f*′ increases as the sample size increases. It can be shown that this criterion for controlling the type-I error, when applied multivariately to equal numbers of cases and controls, gives rise to a smaller type-I error rate once one or more *β*'s are non-zero; see Text S1.

For the DE prior *f*′(*β*) = *ξ* for all *β*>0, thus to control the type-I error at *α* we assign *ξ* to equal the right hand side of (7). For the NEG prior, the value of *f*′ (*β*) is given in (5). The NEG has two parameters, whereas (7) imposes only one constraint. We considered a range of values for the shape parameter *λ* from 0.01 to 10 and then assign *γ* by substituting (7) into (5) and rearranging.

Both the NEG and DE behave similarly when they have been set to have the same type-I error, when their derivative at the origin is the same, and when *β* = 0. Solutions diverge however once SNPs are included in the model, since included SNPs are penalised less by the NEG than by the DE. This results in larger parameter estimates using the NEG, and affects how likely a variable is to be pushed out of the model once it has been included.

### Including Dominant and Recessive Effects

So far, the genotype variable *x_ij_* is the allele count, standardised to have mean zero and variance one. This corresponds to a model that is additive on the logistic scale. To implement a search for dominant or recessive effects, we simply recode this variable accordingly. For example, to seek a recessive effect, we assign *x_ij_* = −*u* if individual *i* is heterozygote or major-allele homozygote at SNP*j*, and *x_ij_* = *v* otherwise, where *u* and *v* are chosen to standardise *x_ij_*. When dominant and recessive effects were included in the model they were considered in the following order: (1) additive, (2) dominant, (3) recessive; terms (2) and (3) are only considered if no preceding term is already included in the model at that SNP.

### Simulation Study

The allelic risk ratios were multiplicative within and across loci and the disease prevalence was 12%; the multiplicative disease model is similar to, but not the same as, the logistic regression model on which our analyses are based. Two causal SNPs were chosen with each of the following approximate MAF values: 2%, 5%, and 15%. The two allelic risk ratios for each MAF were chosen so that the power to detect an association was around 25% and 75% using the ATT at a significance threshold of 10^−5^, see Table 2 for the effect sizes. With this disease model, the background disease risk is typically ≈6% for individuals carrying no causal alleles, and this risk can be attributed either to polygenic or environmental effects. Thus, although we explicitly simulate six causal alleles, this does not exclude multiple weaker causal alleles that are unlikely to be detected.

Marker SNPs were sampled randomly from disjoint 5 Kb regions on each chromosome with probability proportional to MAF(1–MAF), resulting in an approximately uniform MAF distribution.

## Supporting Information

Text S1 Simultaneous Analysis of all SNPs in a Genome-Wide Association Study.
(0.07 MB PDF)
