# Supplementary material for: Simultaneous Analysis of All SNPs in Genome-Wide and Re-Sequencing Association Studies
Source: PLoS Genet. 2008 Jul 25;4(7):e1000130. doi: 10.1371/journal.pgen.1000130 (PMC2464715; doi:10.1371/journal.pgen.1000130)
Supplement: Text S1 — Simultaneous Analysis of all SNPs in a Genome-Wide Association Study. (0.07 MB PDF) [file pgen.1000130.s001.pdf]

Simultaneous Analysis of all SNPs in a Genome–Wide association study – Supplementary Material

Clive J. Hoggart, John C. Whittaker, Maria De Iorio and David J. Balding

May 28, 2008

1 The Normal-exponential-gamma distribution

The normal exponential gamma (NEG) distribution with shape parameter λ and scale parameter γ has probability density function

$$\mathrm{NEG}(\beta \mid \lambda, \gamma) = \kappa \exp\left\{\frac{\beta^2}{4\gamma^2}\right\} D_{-(2\lambda+1)}\left(\frac{|\beta|}{\gamma}\right) \tag{1}$$

where $D_v(z)$ is the parabolic cylinder function and $\kappa = \frac{2^\lambda \lambda}{\gamma\sqrt{\pi}}\Gamma\left(\lambda+\frac{1}{2}\right)$. The derivatives of the NEG density can be calculated from [1]

$$\int_0^\infty x^{\nu-1}(x+\beta^*)^{-\nu+\frac{1}{2}} \exp\{-\mu x\}\, dx = 2^{\nu-\frac{1}{2}}\Gamma(\nu)\mu^{-\frac{1}{2}} \exp\{\beta^*\mu/2\} D_{1-2\nu}\left(\sqrt{2\beta^*\mu}\right) \tag{2}$$

$$\int_0^\infty x^{\nu-1}(x+\beta^*)^{-\nu-\frac{1}{2}} \exp\{-\mu x\}\, dx = 2^{\nu}\Gamma(\nu)\beta^{*-\frac{1}{2}} \exp\{\beta^*\mu/2\} D_{-2\nu}\left(\sqrt{2\beta^*\mu}\right). \tag{3}$$

The first derivative is obtained as follows:

$$\frac{d}{d\beta}\mathrm{NEG}(\beta \mid \lambda, \gamma) = \kappa \frac{d}{d\beta} \exp\left\{\frac{\beta^2}{4\gamma^2}\right\} D_{-(2\lambda+1)}\left(\frac{|\beta|}{\gamma}\right).$$

Substituting $\nu = \lambda + \frac{1}{2},\ \mu = \beta^2/2,\ \beta^* = 1/\gamma^2$ into (3) and rearranging we get

$$= \frac{\kappa}{2^{\lambda+\frac{1}{2}}\Gamma(\lambda+\frac{1}{2})\gamma} \frac{d}{d\beta} \int_0^\infty x^{\lambda-\frac{1}{2}} \left(x+\frac{1}{\gamma^2}\right)^{-(\lambda+1)} \exp\left\{-\frac{1}{2}\beta^2 x\right\} dx$$

$$= -\frac{\kappa}{2^{\lambda+\frac{1}{2}}\Gamma(\lambda+\frac{1}{2})\gamma} \beta \int_0^\infty x^{\lambda+\frac{1}{2}} \left(x+\frac{1}{\gamma^2}\right)^{-(\lambda+1)} \exp\left\{-\frac{1}{2}\beta^2 x\right\} dx$$

and substituting $\nu = \lambda + \frac{3}{2},\ \mu = \beta^2/2,\ \beta^* = 1/\gamma^2$ into (2) and simplifying, we obtain

$$= -\kappa \frac{2\mathrm{sign}(\beta)\left(\lambda+\frac{1}{2}\right)}{\gamma} \exp\left\{\frac{\beta^2}{4\gamma^2}\right\} D_{-(2\lambda+2)}\left(\frac{|\beta|}{\gamma}\right). \tag{4}$$

Similarly, for the second derivative

$$\frac{d^2}{d\beta^2}\mathrm{NEG}(\beta \mid \lambda, \gamma) = -\kappa \frac{2\mathrm{sign}(\beta)\left(\lambda+\frac{1}{2}\right)}{\gamma} \frac{d}{d\beta} \exp\left\{\frac{\beta^2}{4\gamma^2}\right\} D_{-(2\lambda+2)}\left(\frac{|\beta|}{\gamma}\right)$$

substituting $\nu = \lambda + 1,\ \mu = \beta^2/2,\ \beta^* = 1/\gamma^2$ into (3)

$$
\begin{aligned}
&= -\kappa \frac{2\text{sign}(\beta)\left(\lambda+\frac{1}{2}\right)}{2^\lambda \Gamma(\lambda+1)\gamma^2} \frac{d}{d\beta} \int_0^\infty x^\lambda \left(x + \frac{1}{\gamma^2}\right)^{-(\lambda+\frac{3}{2})} \exp\left\{-\frac{1}{2}\beta^2 x\right\}\, dx \\
&= \kappa \frac{2\text{sign}(\beta)\left(\lambda+\frac{1}{2}\right)}{2^\lambda \Gamma(\lambda+1)\gamma^2} \beta \int_0^\infty x^{\lambda+1} \left(x + \frac{1}{\gamma^2}\right)^{-(\lambda+\frac{3}{2})} \exp\left\{-\frac{1}{2}\beta^2 x\right\}\, dx
\end{aligned}
$$

and substituting $\nu = \lambda + 2,\ \mu = \beta^2/2,\ \beta^* = 1/\gamma^2$ into (2) and simplifying, we obtain

$$
= \kappa \frac{4(\lambda+1)\left(\lambda+\frac{1}{2}\right)}{\gamma^2} \exp\left\{\frac{\beta^2}{4\gamma^2}\right\} D_{-(2\lambda+3)}\left(\frac{|\beta|}{\gamma}\right). \tag{5}
$$

Since $f(\beta) = -\log \text{NEG}(\beta \mid \lambda, \gamma)$, and by substituting in (1), (4) and (5) we obtain

$$
\begin{aligned}
f'(\beta) &= -\frac{\frac{d}{d\beta}\text{NEG}(\beta \mid \lambda,\gamma)}{\text{NEG}(\beta \mid \lambda,\gamma)} = \frac{\text{sign}(\beta)(2\lambda+1)}{\gamma} \frac{D_{-(2\lambda+2)}\left(\frac{|\beta|}{\gamma}\right)}{D_{-(2\lambda+1)}\left(\frac{|\beta|}{\gamma}\right)} \\
f''(\beta) &= \frac{\left(\frac{d^2}{d\beta^2}\text{NEG}(\beta \mid \lambda,\gamma)\right)\text{NEG}(\beta \mid \lambda,\gamma) - \left(\frac{d}{d\beta}\text{NEG}(\beta \mid \lambda,\gamma)\right)}{(\text{NEG}(\beta \mid \lambda,\gamma))^2} \\
&= \frac{4}{\gamma^2}\left((\lambda+1)\left(\lambda+\frac{1}{2}\right)\frac{D_{-(2\lambda+3)}\left(\frac{|\beta|}{\gamma}\right)}{D_{-(2\lambda+1)}\left(\frac{|\beta|}{\gamma}\right)} - \left(\left(\lambda+\frac{1}{2}\right)\frac{D_{-(2\lambda+2)}\left(\frac{|\beta|}{\gamma}\right)}{D_{-(2\lambda+1)}\left(\frac{|\beta|}{\gamma}\right)}\right)^2\right).
\end{aligned}
$$

2 The Likelihood

The log–likelihood and its first and second derivatives are given by

$$
\begin{aligned}
L(\boldsymbol{\beta}) &\equiv \log p(\boldsymbol{y}, \boldsymbol{x} \mid \boldsymbol{\beta}) = -\sum_{i=1}^n \log\left(1 + \exp\{-\eta_i\}\right) && (6) \\
L'(\boldsymbol{\beta}) &\equiv \frac{\partial}{\partial \beta_j} L(\boldsymbol{\beta}) = \sum_{i=1}^n \frac{x_{ij} y_i}{1 + \exp \eta_i} \\
L''(\boldsymbol{\beta}) &\equiv \frac{\partial^2}{\partial \beta_j^2} L(\boldsymbol{\beta}) = -\sum_{i=1}^n x_{ij}^2 \frac{\exp \eta_i}{(1+\exp \eta_i)^2} && (7)
\end{aligned}
$$

where $\eta_i = y_i\left(\beta_0 + \sum_{j=1}^k \beta_{ij} x_{ij}\right)$ and $y \in \{-1, 1\}$ denotes case/control status.

If $\beta_j = 0$ it will remain there if the derivative of the log–posterior at the origin is negative in $|\beta|$, which occurs if

$$
\left|\frac{\partial}{\partial \beta_j} \log p(\boldsymbol{y}, \boldsymbol{x} \mid \boldsymbol{\beta} = 0)\right| < f'(\beta_j = 0^+). \tag{8}
$$

This is bounded above and below as follows

$$-\frac{\sum_{i=1}^{n} I(y_i x_{ij} < 0)|x_{ij}|}{1+\exp \eta_{\min}} + \frac{\sum_{i=1}^{n} I(y_i x_{ij} > 0)|x_{ij}|}{1+\exp \eta_{\max}} < \frac{\partial}{\partial \beta_j} \log p(\boldsymbol{y}, \boldsymbol{x} \mid \boldsymbol{\beta})$$
$$< -\frac{\sum_{i=1}^{n} I(y_i x_{ij} < 0)|x_{ij}|}{1+\exp \eta_{\max}} + \frac{\sum_{i=1}^{n} I(y_i x_{ij} > 0)|x_{ij}|}{1+\exp \eta_{\min}},$$

where $I(E)$ equals one when E is true and zero otherwise. Thus the log–likelihood only needs to be calculated when the absolute values of either of the bounds is greater than $f'(\beta_j = 0^+)$. The bounds must be updated every time $\boldsymbol{\beta}$ changes. Implementation of this bound speeded up the code by approximately a factor of 60.

3 Derivation of formula for type–I error probability

We can choose prior parameters to control the type–I error by assuming asymptotic normality of the likelihood function. The asymptotic null distribution of an MLE is [2]

$$\hat{\beta}_j \sim \mathrm{N}\left(0, \left(-\frac{\partial^2}{\partial \beta_j^2} \log p(\boldsymbol{y}, \boldsymbol{x} \mid \boldsymbol{\beta} = 0)\right)^{-1}\right). \tag{9}$$

By evaluating (7) at the null $\boldsymbol{\beta} = 0$, assigning $\beta_0 = \log(n_1/n_0)$, and with standardised genotype data, (9) can be expressed as

$$\hat{\beta}_j \sim \mathrm{N}\left(0, \frac{n_0 + n_1}{n_0 n_1}\right). \tag{10}$$

Differentiating the log–likelihood defined by (10) and substituting into (8) gives

$$|\hat{\beta}_j| \frac{n_0 n_1}{n_0 + n_1} < f'(\beta_j = 0^+)$$

and thus β_j will remain at the origin if

$$|\hat{\beta}_j| < f'(\beta_j = 0^+) \frac{(n_0 + n_1)}{n_0 n_1}. \tag{11}$$

For equal numbers of cases and controls this simplifies to $|\hat{\beta}_j| < 4f'(\beta_j = 0)/(n_0 + n_1)$. For a per-SNP type–I error rate of α we require the probability of (11) to be $1 - \alpha$ at $\beta_j = 0$. From the distribution of $\hat{\beta}_j$ given in (10) this implies

$$f'(\beta_j = 0^+) = \sqrt{\frac{n_0 n_1}{n_0 + n_1}} \Phi^{-1}(1 - \alpha/2). \tag{12}$$

3.1 Behaviour when $\beta_k \neq 0$

When one or more SNPs are included in the model, i.e. $\beta_k \neq 0$, the null distribution of $\hat{\beta}_j$ (9) becomes

$$\hat{\beta}_j \sim \mathrm{N}\left(0, \left\{\sum_{i=1}^{n} x_{ij}^2 \frac{\exp\eta_i}{(1+\exp\eta_i)^2}\right\}^{-1}\right)$$

and from (8) the criterion for inclusion becomes

$$|\hat{\beta}_j| \sum_{i=1}^{n} x_{ij}^2 \frac{\exp\eta_i}{(1+\exp\eta_i)^2} < f'(\beta_j = 0^+)$$

giving a probability of inclusion of

$$P\left(|\hat{\beta}_j| < \frac{f'(\beta_j = 0^+)}{\sum_{i=1}^{n} x_{ij}^2 \frac{\exp\eta_i}{(1+\exp\eta_i)^2}}\right) = 2\Phi\left(\frac{f'(\beta_j = 0^+)}{\sqrt{\sum_{i=1}^{n} x_{ij}^2 \frac{\exp\eta_i}{(1+\exp\eta_i)^2}}}\right) - 1.$$

Substituting in the value of $f'(\beta_j = 0^+)$ given in (12) and assuming equal numbers of cases and controls this can be expressed as

$$= 2\Phi\left(\sqrt{\frac{\frac{1}{4}\sum_{i=1}^{n} x_{ij}^2}{\sum_{i=1}^{n} x_{ij}^2 \frac{\exp\eta_i}{(1+\exp\eta_i)^2}}}\Phi^{-1}(1-\alpha/2)\right) - 1$$

since the numerator in the square–root is greater than the denominator

$$> 1 - \alpha. \tag{13}$$

Thus the test is now conservative. As more SNPs are included in the model the model fit improves, the log–likelihood (6) will increase and the η_i's will get closer to 0 or 1 and the test will become increasingly conservative. This establishes that inclusion of true positives reduces the false positive rate below that expected under the global null.

3.2 Asymptotic equivalence of the ATT and univariate variable selection via shrinkage priors

Since the ATT is equivalent to a score test [3] the test statistic T' for the general multivariate null hypothesis $\boldsymbol{\beta} = 0$ can be written as [2]

$$T' = \left(L'(\boldsymbol{\beta} = 0)\right)^t \left(-L''(\boldsymbol{\beta} = 0)\right)^{-1} L'(\boldsymbol{\beta} = 0).$$

For a univariate hypothesis this can be simplified to

$$T = \frac{L'(\beta_j = 0 \mid \boldsymbol{\beta}_{-j} = 0)}{\sqrt{-L''(\beta_j = 0 \mid \boldsymbol{\beta}_{-j} = 0)}}.$$

From the asymptotic distribution given in (9) and assuming η is constant for all individuals (no other covariate effects) this can be expressed as

$$T = |\hat{\beta}_j| \sqrt{\frac{\exp \eta}{(1 + \exp \eta)^2} \sum_{i=1}^{n} x_{ij}}$$

Since the terms involving η are constant, the condition on β_j remaining at the origin can be expressed as

$$|\hat{\beta}_j| < \kappa \sqrt{\sum_{i=1}^{n} x_{ij}^2} \tag{14}$$

for some constant κ that controls the type–I error at the desired rate.

Returning to variable selection via shrinkage priors; for normalised data and η constant the criteria for β_j remaining at the origin (11) can be expressed as

$$|\hat{\beta}'_j| < \kappa. \tag{15}$$

where κ is determined by the derivative of the prior at the origin; all priors with the same derivative at the origin will have the same type–I error which can be controlled at the desired rate by appropriate choice of prior parameters. Let $\hat{\beta}'_j, x'_{ij}$ and $\hat{\beta}_j, x_{ij}$ denote the MLE and covariates for normalised and unnormalised data respectively. Since $\beta_j x_{ij} = \beta'_j x'_{ij}$ and $x'_{ij} = \frac{x_{ij}}{\sqrt{\sum_{i=1}^{n} x_{ij}/n}}$, $\hat{\beta}_j = \hat{\beta}'_j \sqrt{\sum_{i=1}^{n} x_{ij}/n}$. Rewriting (15) in terms of unnormalised data we get (14), thus the ATT and univariate variable selection using shrinkage priors, starting the search at the origin, are equivalent when our asymptotic assumptions hold.

References

[1] Gradshteyn I, Ryzik I (1980) Tables of Integrals, Series and Products: Corrected and Enlarged Edition. Academic Press: New York.

[2] Cox DR, Hinkley DV (1974) Theoretical statistics. London: Chapman and Hall.

[3] Sasieni PD (1997) From genotypes to genes: doubling the sample size. Biometrics 53:1253–61.
